# Advances in respiratory support for high risk newborn infants

**DOI:** 10.1186/s40748-015-0014-5

**Published:** 2015-05-21

**Authors:** Eduardo Bancalari, Nelson Claure

**Affiliations:** Division of Neonatology, Department of Pediatrics, University of Miami Miller School of Medicine, Miami, Florida USA

**Keywords:** Mechanical ventilation, Supplemental oxygen, Premature infant, Advances

## Abstract

**Background:**

A significant proportion of premature infants present with respiratory failure early in life and require supplemental oxygen and some form of mechanical respiratory support.

**Findings:**

Many technical advances in the devices for neonatal respiratory support have occurred in recent years and new management strategies have been developed and evaluated in this population. This article describes some of these novel methods and discusses their application and possible advantages and limitations.

**Conclusion:**

Newer methods of respiratory support have led to marked improvement in outcome of premature infants with respiratory failure. Some of these strategies are very promising but further investigation to evaluate their short term efficacy and impact on long term respiratory and other relevant outcomes is needed before wider use.

## Introduction

Different modes of respiratory support and oxygen supplementation are commonly used in premature infants in respiratory failure. The strategies of respiratory support and the devices utilized for this purpose have evolved considerably. Since the earlier studies describing the use of intermittent positive pressure ventilation, the advantages of continuous distending pressure in the form of nasal **C**PAP, the use of the T piece to provide a continuous flow of gas from which the patient can breathe spontaneously and the application of positive end-expiratory pressure [1-10] have constituted the basis of the modern neonatal respiratory support. These, combined with the introduction of therapies such as antenatal steroids and exogenous surfactant, have produced improvements in survival of high risk premature infants in respiratory failure.

The respiratory outcome of these high risk infants has also improved considerably compared to the severe lung injury induced by high positive pressure and elevated inspired oxygen observed during the early years of respiratory support in premature infants [11-13]. These improvements resulted from a better understanding of the damage associated with the aggressive use of mechanical ventilation and high inspired oxygen levels to maintain normal arterial blood gases and achieve control of the infant’s ventilation [14-16]. Current strategies of neonatal respiratory support aim to produce adequate gas exchange while minimizing the risk of lung injury and by facilitating weaning with ventilator strategies that primarily assist the infant’s spontaneous respiratory effort. More recent advances seek to adjust the different forms of respiratory support to the infant’s changing needs and further facilitate their spontaneous breathing and minimize the risk of lung injury. These include advances in monitoring technology and automation of specific parameters of the respiratory support.

## Review

### Synchronized mechanical ventilation

The use of the T piece and the circulating bias flow in neonatal mechanical ventilators to maintain PEEP and provide cycles where the pressure increases to the set peak inspiratory pressure (PIP) level at fixed intervals became known as time-cycled pressure-limited (TCPL) ventilation. The intermittent tidal inflation during TCPL is also known as intermittent mandatory ventilation (IMV).

During IMV the clinician sets the PIP and cycling frequency depending on the level of ventilatory support required by the infant. The cycling frequency is gradually reduced as the infant contribution to minute ventilation increases. During this process there is continuous interaction between the infant’s spontaneous breathing and the ventilator positive pressure cycles. At times, ventilator cycles can interfere with the infant’s breathing when they occur late in the infant’s spontaneous inspiration or during exhalation.

**Figure 1:**
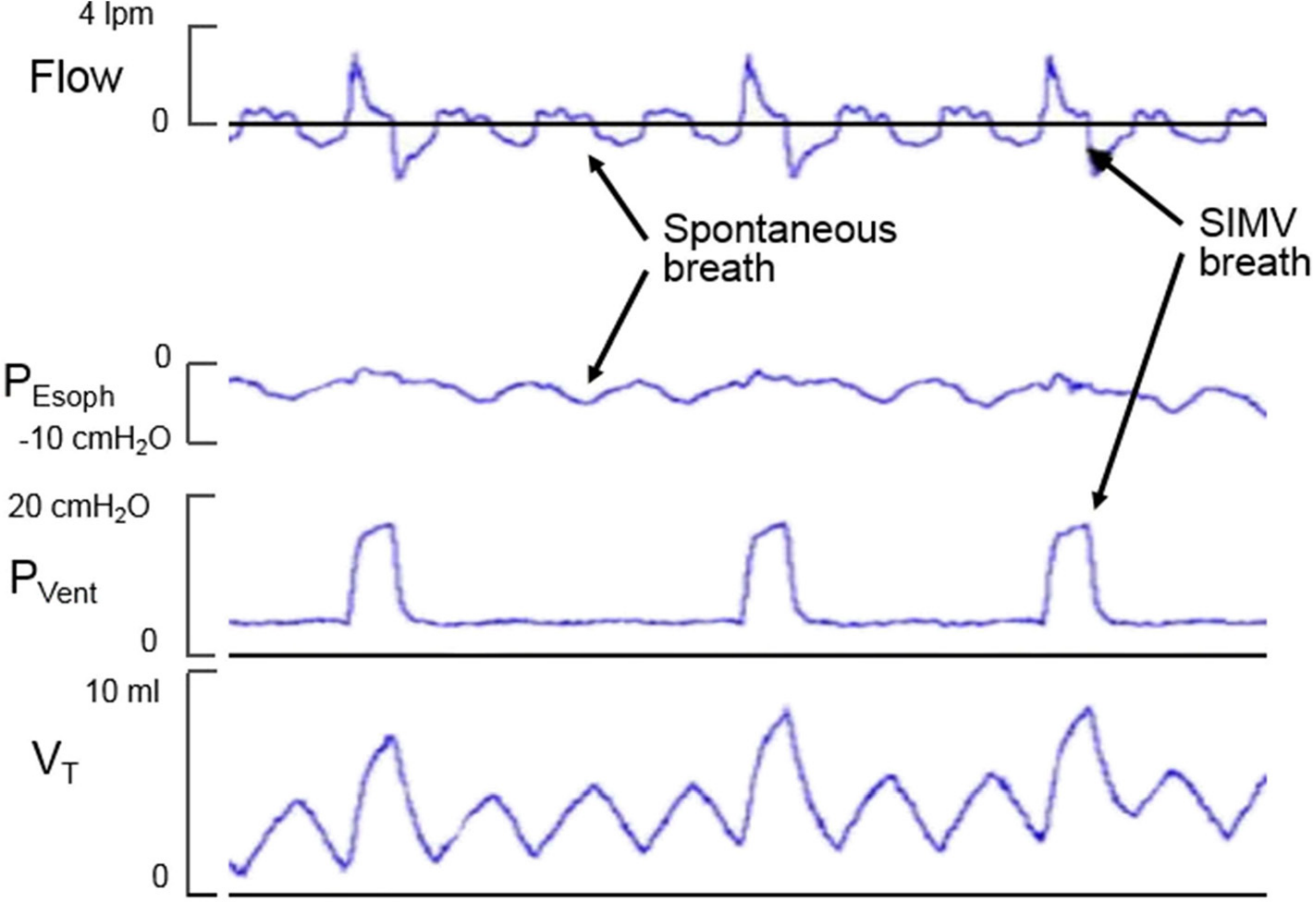
Synchronized intermittent mandatory ventilation. Tracings of flow, esophageal pressure (P_Esoph_), ventilator pressure (P_Vent_) and tidal volume (V_T_) obtained from a premature infant undergoing SIMV. The interval between SIMV cycles is adjusted by the ventilator to maintain synchrony with the infant’s inspiratory efforts (negative deflections in the esophageal pressure). The combination of the positive pressure and the infant’s spontaneous inspiratory effort achieve a larger tidal volume than that of the non-assisted spontaneous breaths.

The use of various sensors in neonatal ventilators to detect the infant’s spontaneous inspiration can achieve synchronized delivery of the positive pressure cycles with the onset of the infant’s inspiration. This resulted in the development of various modalities of support including synchronized IMV (SIMV), assist/control (A/C) and pressure support ventilation (PSV) [17-22]. The tracing in Figure 1 shows representative recordings from a premature infant undergoing SIMV.

## Findings

Clinical studies have shown that the addition of the pressure generated by the infant’s respiratory pump and that produced by the ventilator during synchronized ventilation result in a more consistent tidal volume and improved ventilation compared to IMV, leading to a more stable and effective gas exchange [23-29]. Clinical trials have consistently shown faster weaning and shorter duration of mechanical ventilation with synchronized modes compared to IMV which is more evident among the more premature infants [30-35]. These studies underline the importance of preserving the infant’s spontaneous breathing and provide only the necessary level of support to assist ventilation better than controlling the infant’s ventilation and gas exchange.

In spite of the reduction in duration of mechanical ventilation the effects on respiratory outcome, namely bronchopulmonary dysplasia (BPD), have not been consistent. These however appear to be more striking in those studies enrolling more immature infants at higher risk of BPD [36]. As described, the reduction in BPD by synchronized ventilation is greatest in those studies where the study population had a higher rate of BPD.

**Figure 2:**
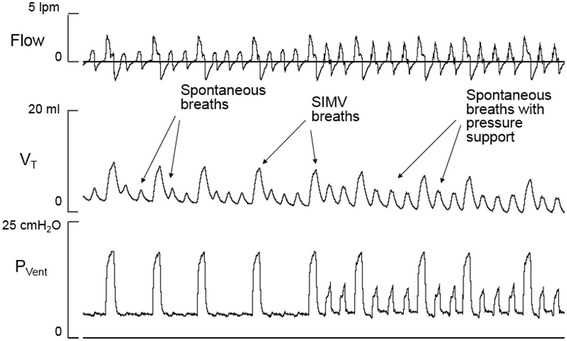
Synchronized intermittent mandatory ventilation combined with pressure support. Tracings of flow, tidal volume (V_T_) and ventilator pressure (P_Vent_) obtained from a premature infant switched from SIMV to SIMV combined with pressure support. The tidal volume from spontaneous breaths assisted by pressure support is larger than the tidal volume from non-assisted spontaneous breaths between SIMV cycles.

Studies have not shown clear advantages of one synchronized modality versus others except for a slightly faster weaning with A/C [37,38]. The similarity of the effect is likely due to the fact that these modalities provide comparable levels of support during the initial phase of acute respiratory distress where higher rates used in SIMV provide similar support as A/C or PSV. One study showed the additional use of PSV to SIMV can facilitate the weaning compared to SIMV alone. Lower peak pressure levels with PSV boosted the spontaneous breaths reducing the reliance on larger SIMV breaths [39]. Figure 2 shows a representative recording from an infant receiving SIMV combined with pressure support.

Synchronized modalities of neonatal ventilation can be rendered ineffective if the breath sensing parameters are not set properly to detect the infant’s spontaneous inspiration. When this occurs the infant receives only mandatory breaths and can experience the consequent asynchrony. The most important concern with synchronized modalities is the risk of autocycling when artefacts or a too sensitive threshold for synchrony can result in the ventilator providing a cycle that is not necessarily triggered by a spontaneous inspiration. This is a greater concern in modes such as A/C and PSV when there is a risk of hypocapnia or gas trapping because of autocycling at very high ventilator rates [40].

### Monitoring during mechanical ventilation

For many years the adequacy of ventilation was limited to monitoring of blood gases, radiographic evaluation, visual assessment of chest expansion and monitoring of breathing frequency by transthoracic impedance. The introduction of flow sensors for synchronization also provided the clinician with the ability to monitor the adequacy of tidal and minute volume. This led to a more objective evaluation of the tidal inflation and to a better titration of PIP which may be associated with reduced lung injury [41-43].

### Non-invasive respiratory support

In recent years non-invasive respiratory support by nasal continuous positive airway pressure (NCPAP) and nasal intermittent positive pressure ventilation (NIPPV) have being increasingly used in the premature infant instead of mechanical ventilation. The effects of the application of a continuous distending pressure with NCPAP include stabilization of lung volume and airway patency leading to improved oxygenation and reduced apnea. NIPPV may enhance the effects of NCPAP by increasing ventilation and mean airway pressure, by washing out of CO2 from the upper airway and by a possible enhancement of the respiratory drive.

### Findings

Clinical studies in preterm infants after extubation to NIPPV showed increased ventilation and reduced PaCO_2_ and breathing effort [44,45]. In more stable infants NIPPV did not increase ventilation or improve gas exchange but it reduced breathing effort compared to NCPAP [46-48]. This suggests a greater benefit of NIPPV over NCPAP in infants with some degree of ventilatory failure or those struggling to maintain adequate ventilation. Although NIPPV has not been consistently shown to be more effective than N-CPAP in reducing apnea, its efficacy appears to increase when synchronized to the infant’s spontaneous breathing [44,49-52].

Randomized controlled trials in premature infants with RDS have shown that NIPPV can reduce the need for mechanical ventilation compared to NCPAP [53-58]. The efficacy of synchronized NIPPV in reducing extubation failure compared to NCPAP has been shown consistently in randomized trials [59-63]. Although NIPPV is more effective than NCPAP in reducing the need for mechanical ventilation, studies have not demonstrated a significant impact of NIPPV compared to NCPAP on pulmonary outcome [53-64]. These clinical trials did not show increased risk of side effects such as serious gastrointestinal gas distension or pulmonary air leaks.

Although the use of both NCPAP and NIPPV has become very common, there is considerable need to further develop and test newer technologies to improve patient interfaces, synchronization of the ventilator with the patient and to improve transmission of the positive pressure to the infant’s airways.

### Automation of respiratory support

The peak pressure during each ventilator cycle set by the clinician provides a constant level of ventilatory support. However, the ventilatory needs of the premature infant in respiratory failure can vary considerably within short periods of time. These fluctuations in respiratory mechanics and spontaneous breathing effort can lead to significant variations in ventilation. Because the support level provided by ventilatory modes such as SIMV, IMV, A/C or PSV provide constant peak pressure and/or mandatory rate, the set levels generally exceed those required by premature infants in order to maintain adequate ventilation at all times, even at times when the infant’s needs may be less. In order to adjust the ventilatory support to the infant’s needs, methods for automatic adjustment of the peak pressure and frequency have been incorporated to neonatal ventilators.

### Volume targeted ventilation

**Figure 3:**
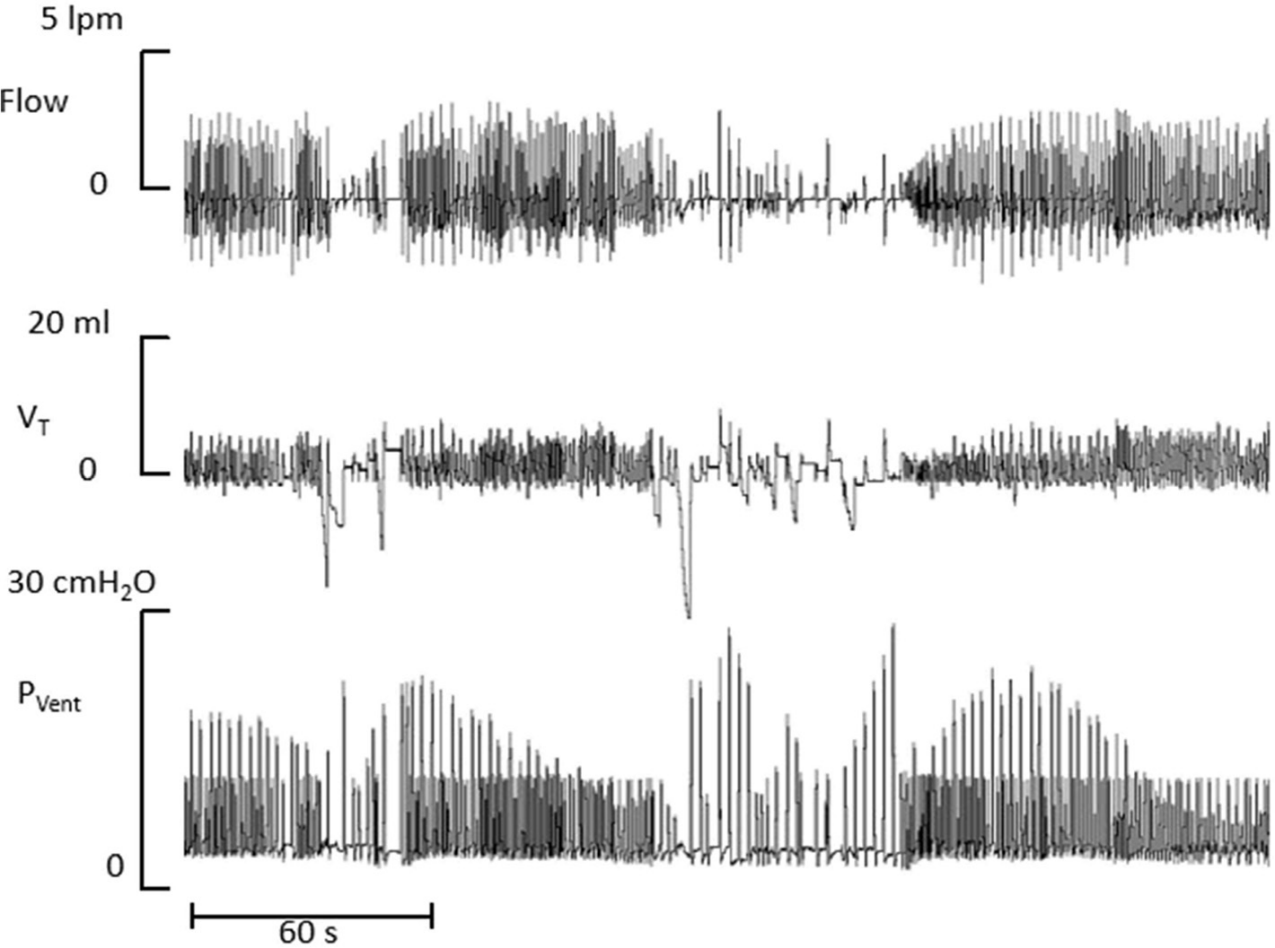
Volume guarantee ventilation. Tracings of flow, tidal volume (V_T_) and ventilator pressure (P_Vent_) from a premature with frequent fluctuations in ventilation. The ventilator peak pressure is automatically increased to maintain tidal volume at the target level during periods of instability and it declines as ventilation becomes more stable.

During volume targeted ventilation, the peak pressure is automatically adjusted to maintain the tidal volume at the level set by the clinician. In this manner, when respiratory mechanics improve or the infant’s respiratory pump produces a larger tidal volume the ventilator reduces the peak pressure provided in each cycle and vice-versa. The adjustments in ventilator pressure are expected to provide better ventilation stability. Figure 3 shows a representative recording from a premature infant receiving volume guarantee ventilation.

During volume targeted ventilation the ventilator can only control the peak positive pressure applied on each cycle but cannot determine the negative pressure produced by the infant’s respiratory pump. The targeted volume in this modality is essentially a minimum tidal volume level but does not prevent the spontaneous inspiratory effort from generating breaths that exceed the target level.

### Findings

Clinical studies have shown improved stability and reduced exposure to excessive or insufficient tidal volumes and an effective automatic reduction in PIP [65-67]. Clinical trials have demonstrated volume targeted ventilation modes can facilitate weaning from mechanical ventilation compared to conventional manual titration of peak pressure [68-72]. The combined data from these studies shows a significant reduction in the rate of the composite outcome BPD or death [73].

Although volume targeted ventilation appears to be safe there are important aspects to be considered in regards to its efficacy. Different ventilators utilize various methods to achieve volume targeted ventilation. Some of the ventilators make the automatic adjustments in pressure based on the volume delivered by the ventilator to the breathing circuit while others take the tidal volume measured with proximal flow sensors or from an estimate of the tidal volume from measurements obtained by flow sensors built in the ventilator. Ventilators also vary on whether the adjustments in pressure are made based on the tidal volume measured during the inspiratory phase or exhaled volume or in the timing of the pressure adjustment, i.e. instantaneously as the volume is delivered or from one cycle to the next.

Although there is sufficient evidence to recommend avoidance of extreme high or low tidal volumes, there is insufficient information in regards to the optimal tidal volume to be used in infants of different gestational ages, indications for mechanical ventilation and during the different phases of respiratory failure. Data are also lacking on the most adequate tidal volume target when volume targeted ventilation is provided at different mandatory rates with SIMV or when it is used to assist every spontaneous inspiration in A/C or PSV.

### Targeted minute ventilation

The ventilatory support provided during conventional ventilation at a frequency set by the clinician provides a relatively constant minimum level of minute ventilation. This level of support may be adequate for most of the time but at times it can be insufficient and/or excessive to meet the infant’s needs. This is because the contribution of the infant’s spontaneous breathing effort to the total minute ventilation varies considerably depending on the consistency of the respiratory pump, stability of respiratory mechanics and the infant’s respiratory drive.

Targeted minute ventilation consist of automatic adjustments of the cycling frequency of the ventilator to maintain the minute ventilation at a level set by the clinician or alternatively to keep the total respiratory rate at a preset level. Because the ventilator can only control its own cycling frequency and not the infant’s, these modalities only target a minimum level of minute ventilation or respiratory rate.

### Findings

In short term clinical studies in preterm infants these automatic modalities have been shown to be effective in reducing the ventilator frequency without affecting gas exchange [74,75]. Modalities such as PSV can be used to assist every spontaneous inspiration and in the event of apnea the ventilator provides mandatory cycles at a frequency and PIP set by the clinician. The effects of PSV as a stand- alone mode and the impact of the back-up ventilation provided during apnea have not been evaluated in preterm infants. Other methods include automatic adjustments to the cycling frequency not only when ventilation declines but also in response to decreases in arterial oxygen saturation [76].

Although these modalities are promising alternatives to tailor the respiratory support to the changing needs of the infant, to date there have not been randomized trials evaluating their efficacy in improving respiratory outcome in this population.

Newer experimental developments include simultaneous adjustments of both the cycling frequency and the peak pressure of the ventilator. The combined approach was more effective than conventional pressure ventilation or the individual automatic adjustment of pressure or frequency in maintaining oxygenation in an animal model of induced episodic hypoxemia [77].

### Proportional assist ventilation and neurally adjusted ventilatory assist

In proportional assist ventilation (PAV) the ventilator assists the infant’s respiratory pump to overcome elastic or resistive loads due to the underlying lung disease. For this, the ventilator pressure is automatically adjusted in proportion to the measured tidal volume, flow or both. The proportionality factor set by the clinician determines the degree of unloading or compensation for the disease induced respiratory loads. When PAV is used the infant essentially perceives his respiratory mechanics have improved because of the simultaneous provision of positive pressure as the infant generates each inspiratory effort.

### Findings

In clinical studies PAV has been shown to be effective in reducing the inspiratory effort and providing ventilation with lower ventilator pressures compared to conventional modes in premature infants recovering from respiratory failure or with evolving chronic lung disease [78,79].

**Figure 4:**
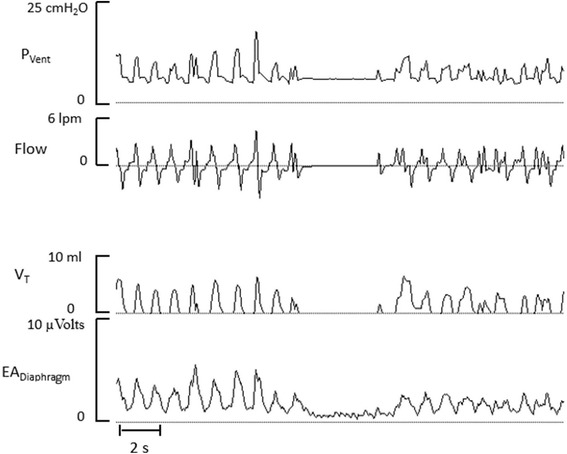
Neurally adjusted ventilatory assist. Tracings of ventilator pressure (P_Vent_), flow, tidal volume (V_T_) and diaphragmatic electrical activity (EA_Diaphragm_) from a premature undergoing NAVA. The ventilator pressure is proportional to the magnitude of the electrical activity of the diaphragm during each inspiration. Note the absence of support during a breathing pause. A longer pause would require a backup rate of ventilator cycles.

Neurally adjusted ventilatory assist (NAVA) is a modality where the ventilator pressure is automatically adjusted in proportion to the measured electrical activity of the diaphragm. NAVA is intended to enhance the infant’s ability to generate V_T_ and/or reduce the diaphragm’s activity. Figure 4 shows a representative recording from a premature infant undergoing NAVA.

### Findings

In short term studies NAVA has been shown to maintain similar or better ventilation and gas exchange with lower pressures and better synchrony compared to conventional ventilation in preterm infants, but without a significant reduction in diaphragmatic activity [80-83].

PAV and NAVA are promising alternatives but their long term effects need to be explored to determine their impact on weaning from mechanical ventilation and on pulmonary outcomes in high risk preterm infants.

### Automatic control of inspired oxygen

Most preterm infants in respiratory failure or with chronic lung disease require supplemental oxygen but because of their prematurity they are at risk of damage to their eyes and other organs if the arterial oxygen levels are excessive or insufficient [84,85]. Although arterial oxygen saturation levels are continuously monitored by pulse oximetry (SpO_2_) these infants spend considerable periods of time outside the intended prescribed range [86,87]. While fluctuations below the targeted range of oxygenation are usually episodic and due to the infant’s respiratory instability, high SpO_2_ levels in oxygen dependent infants are generally induced by excessive fraction of inspired oxygen (FiO_2_) [88-90].

**Figure 5:**
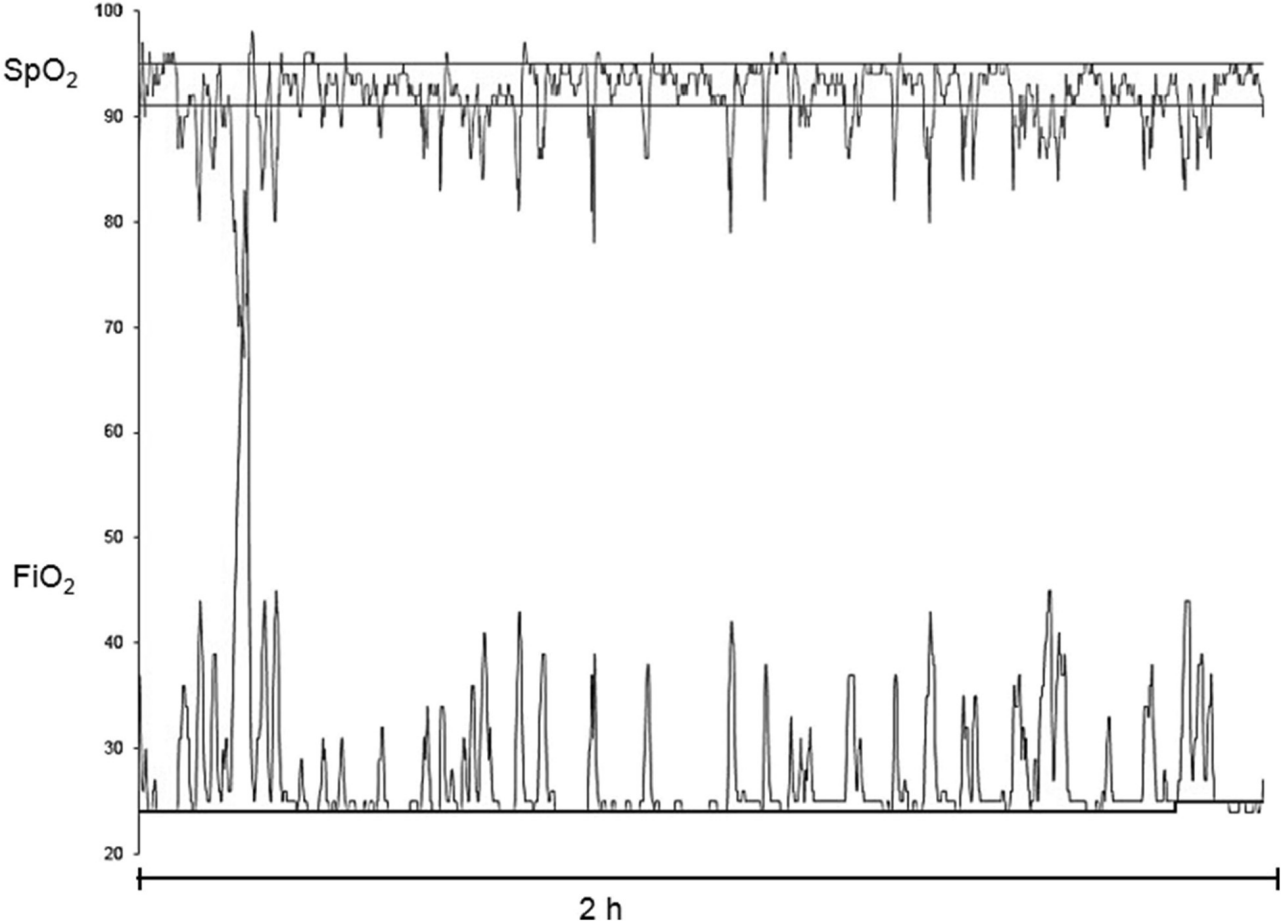
Automatic control of inspired oxygen. Recordings of SpO_2_ and FiO_2_ from a premature infant during 2 hours of automatic control of FiO_2_. The ventilator automatically increases FiO_2_ when SpO_2_ declines below the target in order to keep SpO_2_ within the target range (horizontal lines). The magnitude of the increases in FiO_2_ are proportional to the decline in SpO_2._ FiO_2_ is brought back to the baseline level (thicker horizontal line) after SpO returns to the target range.

Systems for automatic control of FiO_2_ have been developed with the goal of improving the maintenance of SpO_2_ within a target range and consequently reduce exposure to hyperoxemia and supplemental oxygen as well as to attenuate episodes of hypoxemia. Figure 5 shows representative recordings of SpO_2_ and FiO_2_ from a premature infant undergoing automatic control of FiO_2_.

### Findings

In short term clinical studies these systems have been shown to be more effective than manual adjustments by the routine staff and by a fully dedicated nurse at bedside in keeping SpO_2_ within the target range [91-104]. In these studies the reduction in hyperoxemia and oxygen exposure was also significant particularly in premature infants with frequent fluctuations in oxygenation. In these infants the clinical staff often tolerates SpO_2_ levels well above the intended range to prevent or attenuate the episodes of hypoxemia.

Although the systems of automatic control of FiO_2_ have shown promising results, their impact on longer term ophthalmologic, respiratory and neurologic outcome still remains to be determined in large scale clinical trials.

These automated systems are intended to replace the repetitive task of manual titration of FiO_2_ and in this way enhance the efficacy of the clinical staff. The clinical studies mentioned above have shown a striking reduction in the number of manual FiO_2_ adjustments by the clinical staff during automatic FiO_2_ control. While this is a positive finding, these systems are not a substitute for continuous clinical observation of the patient and should not reduce attentiveness of the caregiver. The ability to integrate monitoring of SpO_2_ and the need for FiO_2_ into smarter alarms and warnings can mitigate potential situations when automatic adjustments could mask a respiratory deterioration and may also enhance the care since FiO_2_ levels are not commonly monitored at present time.

These automatic systems aim to maintain a range of SpO_2_ set by the clinician. However, at this time there is no consensus on the most appropriate target range of SpO_2_ for premature infants due to the conflicting and competitive clinical outcomes of different target ranges. In some trials lower oxygenation target ranges appear to reduce severe retinopathy of prematurity and BPD but also appear to reduce survival of the extremely premature infant [105-107]. It is also important to note that target ranges of SpO_2_ in observational and interventional randomized clinical trials were not closely matched by the actual SpO_2_ levels. Therefore caution is recommended when setting the target range in an automatic system as this would maintain such range more closely than the routine care. This may uncover effects of different target ranges that were not previously observed simply because the maintenance of SpO_2_ within such range was not adequate.

## Conclusion

In summary, advances in the devices and new strategies to provide invasive and non-invasive respiratory support have achieved considerable improvements in the management of the critically ill premature infant. Further investigation to evaluate their short and long term efficacy and impact on respiratory and other relevant clinical outcomes is needed.

## Abbreviations

CPAP: Continuous positive airway pressure
PEEP: Positive end-expiratory pressure
PIP: Peak inspiratory pressure
TCPL: Time-cycled pressure-limited
IMV: Intermittent mandatory ventilation
SIMV: Synchronized intermittent mandatory ventilation
A/C: Assist/control
PSV: Pressure support ventilation
NCPAP: Nasal continuous positive airway pressure
NIPPV: Nasal intermittent positive pressure ventilation
BPD: Bronchopulmonary dysplasia
RDS: Respiratory distress syndrome
PAV: Proportional assist ventilation
NAVA: Neurally adjusted ventilatory assist
SpO_2_: Arterial oxygen saturation pulse oximetry
FiO_2_: Fraction of inspired oxygen
